# Functional and structural analysis of partial optic nerve avulsion due to blunt trauma: Case report

**DOI:** 10.4103/0301-4738.71705

**Published:** 2010

**Authors:** Tarkan Mumcuoglu, Hakan A Durukan, Cuneyt Erdurman, Volkan Hurmeric, Fatih C Gundogan

**Affiliations:** Department of Ophthalmology, Gulhane Military Medical School, Etlik, Ankara, Turkey

**Keywords:** Blunt ocular trauma, optical coherence tomography, optic nerve avulsion

## Abstract

Partial optic nerve avulsion (ONA) secondary to finger gouging is an uncommon but devastating injury. A 21-year-old man who had an acute vision loss after accidentally getting poked by himself in his right eye when he fell down during jogging is reported. The patient was diagnosed with partial ONA. Magnetic resonance imaging revealed intact optic nerve. Optical coherence tomography (OCT) revealed deep cavity at the inferior-temporal half of the optic disc. Retinal nerve fiber layer thickness was also thin at the inferior quadrant with circumpapillary OCT scan. Visual field test and electrophysiological tests showed functional abnormality compatible with optic nerve lesion. Diagnostic tools for anatomical and functional evaluation may reveal the course of this injury.

Optic nerve avulsion (ONA) is defined as a traumatic disinsertion of the nerve fibers at the disc margin but without damage to the disc sheath and is an uncommon form of traumatic optic neuropathy.[1] ONA is part of anterior traumatic optic neuropathy and forceful rotation of the globe is the mechanism of the disease. This report highlights the risk for severe vision loss resulting from finger gouging and novel diagnostic methods for its evaluation.

## Case Report

**Figure 1:**
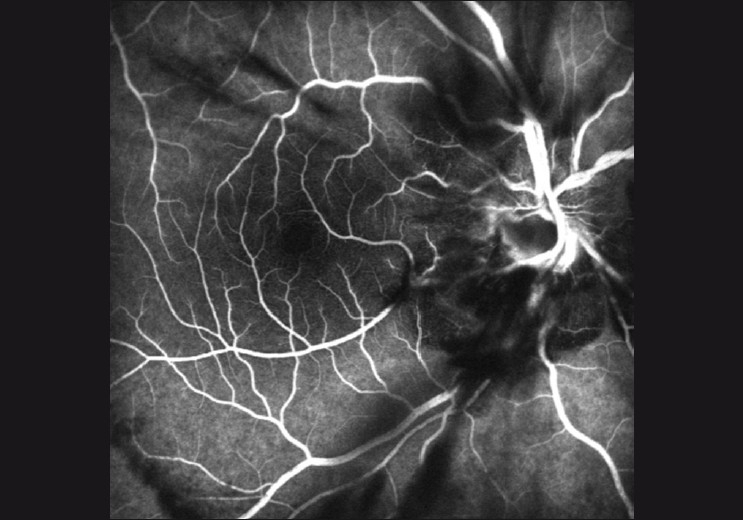
Fluorescein angiography showing peripapillary subretinal hemorrhages and vitreous hemorrhage at the first examination

**Figure 2:**
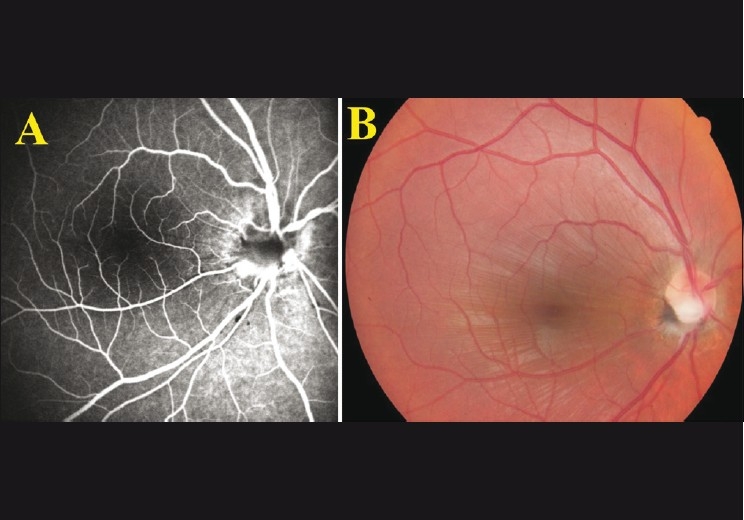
(A) Fluorescein angiography and (B) fundus photo revealing choroidal folds and partial optic nerve avulsion on the 20^th^ day after the trauma

A 21-year-old man presented with acute vision loss immediately following his own finger gouging of his right eye while accidentally falling down during jogging. Visual acuity was counting fingers at 1 meter in the right eye and 20/20 in the left. The left eye was normal. A relative afferent pupillary defect was noted in the right eye. Intraocular pressure was 12 mmHg. Slit-lamp examination revealed a subconjunctival hemorrhage affecting the temporal aspect of the right eye. The cornea and lens were intact and clear. Dilated fundus examination revealed peripapillary subretinal hemorrhages and vitreous hemorrhage most prominently in the inferior vitreous cavity. Mild peripapillary edema and choroidal folds were observed. Fluorescein angiography revealed the masking of fluorescence due to intravitreal hemorrhage around the optic disc [Fig. 1]. The patient was treated with a 20-day course of tapering systemic steroid (prednisolone, 64 mg, peroral). Subsequently, vitreous hemorrhage settled and partial ONA at the lower half of the optic disc was recognized 20 days after the injury [Fig. 2].

Magnetic resonance imaging (MRI) of the orbits showed intact optic nerve bilaterally. But focal contrast enhancement was observed at the right optic nerve.

**Figure 3:**
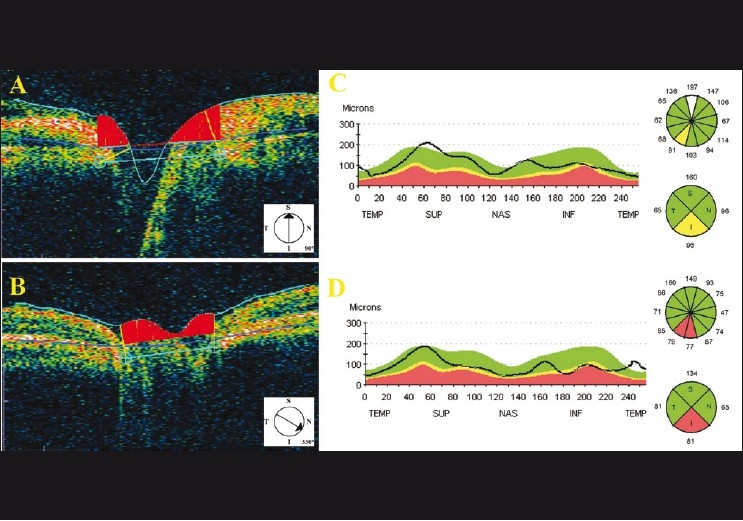
Optical coherence tomography findings of the affected eye. (A) Vertical scan of the optic disc showing deep cavity at the inferiortemporal half. (B) Subsequent scan revealing physiological cupping without any cavity. Circumpapillary scans on Days 30 (C), and 75 (D) after injury

For structural evaluation, optical coherence tomography (OCT; Stratus OCT, Carl Zeiss Meditec, Dublin, CA) was performed after one month from the injury. Cross-sectional vertical scan from inferior to superior quadrant of the optic disc with OCT signal strength 7 revealed deep cavity at the inferior-temporal half [Fig. 3A and B]. Circumpapillary scan showed thin inferior retinal nerve fiber layer (RNFL) with a thickness of 96 microns with respect to OCT normative database in the thickness profile graph and quadrant analysis [Fig. 3C]. RNFL thickness of the fellow eye was 133 microns for the inferior quadrant, 81 microns for the temporal quadrant, 150 microns for the superior quadrant, and 94 microns for the nasal quadrant. The analysis with the comparison to the normative dataset showed a borderline thin RNFL at the 7:00 position in the clock hour analysis and at the inferior quadrant [Fig. 3C]. Circumpapillary RNFL measurement at 75 days after trauma revealed an abnormally thin RNFL at the 6:00 and 7:00 positions in the clock hour analysis. Quadrant analysis was also apparently abnormal in the inferior part with the thickness of 81 microns [Fig. 3D].

**Figure 4:**
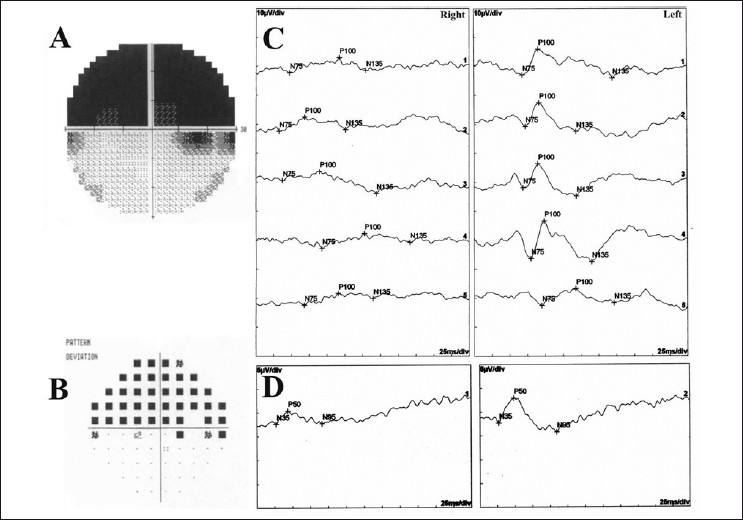
(A, B) Automated perimetry showing superior altitudinal visual field defect on the gray-scale and pattern deviation probability map. (C) Pattern visual evoked potentials testing showing even unrecordable P100 peaks. (D) Pattern electroretinogram testing showing reduced P50 peak amplitude in the right eye

Visual field test and electrophysiological tests were performed for functional evaluation. Humphrey visual field testing revealed a superior altitudinal visual field defect [Fig. 4A and 4B]. Monocular pattern visual evoked potentials (PVEP) testing to five different check sizes (2’, 1’, 30°, 15°, 7°) showed even unrecordable P_100_ peaks to all checks in the right eye. Transient pattern electroretinogram (PERG) testing showed reduced P_50_ peak amplitude in the right eye [Fig. 4C and 4D].

Vision acuity was 20/200 at the last visit. Fundus photo and angiography revealed inferiorly partial ONA, peripapillary atrophy, and choroidal folds at the macula.

## Discussion

ONA is the traumatic separation of the optic nerve from the globe at the level of the lamina cribrosa without rupture of the optic nerve sheath or the adjacent sclera.[2] ONA results in partial or permanent loss of vision in the affected eye. ONA may result from a blunt injury where a foreign object intrudes between the globe and the orbital wall.[1,3] ONA is part of anterior traumatic optic neuropathy (anterior TON) and its pathophysiology is rotational movement of the globe around its axis that causes tearing of the optic nerve parenchyma without involving the optic nerve sheath. Another part of anterior TON is direct penetration of a foreign body through the medial part of the orbit into the anterior part of the optic nerve. In this case, the dura may be disinserted as well.

A literature search showed that finger poking is one of the most common causes of injury.[4,5] Cirovic *et al*.[2] showed in their computer modeling study that the point at which the optic nerve inserts into the sclera, on the opposite side to the finger impact, would appear to be under the greatest strain. The authors calculated large strains in the lamina cribrosa. They suggested that there were two principal mechanisms of the injury: the rotation of the optic nerve relative to the globe, and the increased intraocular pressure arising from the deformation of the globe. Buchwald *et al*.[6] reported that the cause of ONA was a small blunt object or finger that strokes the eye in 49% of the patients. They showed that the visual fields in eyes with partial ONA revealed a defect in the superior visual field in six of 12 patients. The case presented in this study also had superior visual field defect.

MRI was shown not to be sensitive enough to independently evaluate ONA.[1,7] Simsek *et al*.[8] suggested B-scan ultrasonography to evaluate ONA and reported that B-scan ultrasonography detected ONA clearly, although MRI evaluation yielded normal results. In contrast, Foster *et al*.[1] reported that ultrasonographic evaluations failed to detect ONA in four cases. Simsek*et al*.[8] concluded that the results of imaging evaluations in ONA were not uniform.

OCT has proved to be a promising technology for assessing the thickness of tissues *in vivo*. OCT was reported to be an important adjunctive tool in cases of posterior segment trauma.[9] Medeiros *et al*.[10] described a case with progressive RNFL loss after acute injury to the optic nerve. They suggested that OCT might be useful to monitor RNFL loss over time. Vessani *et al*.[11] also reported a case of indirect optic nerve injury which had a progressive macular thinning detected by macular scans using OCT. We performed OCT scans with six equally spaced radial planes, each passing through the center of the optic nerve head. Partial ONA was obviously shown by vertical scan through the optic disc at the inferior-temporal area even though the cavity was not observed with the 30 degrees apart consequent scan.

Even unrecordable monocular P_100_ responses in the affected eye showed the significant functional morbidity of ONA in our case. In addition, PERG (a retinal response) showed P_50_ amplitude reduction in our case that indicates the impairment of the macular function. This may result from retinochoroidal folds in the central retina or a primary traumatic maculopathy although the macula had ophthalmologically normal light reflex.

In conclusion, the results of imaging modalities in ONA are not consistent.[8] As a new imaging modality, OCT may clearly detect even partial avulsion.

## References

[1] Foster BS, March GA, Lucarelli MJ, Samiy N, Lessell S (1997). Optic nerve avulsion. Arch Ophthalmol.

[2] Cirovic S, Bhola RM, Hose DR, Howard IC, Lawford PV, Marr JE (2006). Computer modelling study of the mechanism of optic nerve injury in blunt trauma. Br J Ophthalmol.

[3] Hykin PG, Gardner ID, Wheatcroft SM (1990). Optic nerve avulsion due to forced rotation of the globe by a snooker cue. Br J Ophthalmol.

[4] Fard AK, Merbs SL, Pieramici DJ (1997). Optic nerve avulsion from a diving injury. Am J Ophthalmol.

[5] Chow AY, Goldberg MF, Frenkel M (1984). Evulsion of the optic nerve in association with basketball injuries. Ann Ophthalmol.

[6] Buchwald HJ, Spraul CW, Wagner P, Lang GK (2001). Optic nerve evulsion: Meta-analysis. Klin Monatsbl Augenheilkd.

[7] Sawhney R, Kochhar S, Gupta R, Jain R, Sood S (2003). Traumatic optic nerve avulsion: Role of ultrasonography. Eye.

[8] Simsek T, Simsek E, Ilhan B, Ozalp S, Sekercioglu B, Zilelioglu O (2006). Traumatic optic nerve avulsion. J Pediatr Ophthalmol Strabismus.

[9] Rumelt S, Karatas M, Ophir A (2005). Potential applications of optical coherence tomography in posterior segment trauma. Ophthalmic Surg Lasers Imaging.

[10] Medeiros AF, Moura FC, Vessani RM, Susanna R (2003). Axonal loss after traumatic optic neuropathy documented by optical coherence tomography. Am J Ophthalmol.

[11] Vessani RM, Cunha LP, Monteiro ML (2007). Progressive macular thinning after indirect traumatic optic neuropathy documented by optical coherence tomography. Br J Ophthalmol.

